# Cephalometrics of Pharyngeal Airway Space in Lebanese Adults

**DOI:** 10.1155/2017/3959456

**Published:** 2017-01-04

**Authors:** Antoine Daraze, Myriam Delatte, Giuseppe Liistro, Zeina Majzoub

**Affiliations:** ^1^Department of Orthodontics, Lebanese University, School of Dentistry, Beirut, Lebanon; ^2^Department of Orthodontics, Cliniques Universitaires Saint-Luc, Université Catholique de Louvain, Brussels, Belgium; ^3^Department of Pneumology, Cliniques Universitaires Saint-Luc, Université Catholique de Louvain, Brussels, Belgium; ^4^Department of Periodontics, Lebanese University, School of Dentistry, Beirut, Lebanon

## Abstract

*Purpose*. The upper airway space is significant in orthodontic diagnosis and treatment planning. The objectives of this study are to assess the dimensions of soft tissue elements of the upper pharyngeal space and evaluate potential correlations with modifying variables such as gender, skeletal class, and anthropometric parameters.* Materials and Methods*. Lateral cephalograms were obtained from 117 healthy young adult Lebanese subjects. Nineteen cephalometric linear/angular measurements of the nasopharynx, oropharynx, and hypopharynx were recorded. Anthropometric parameters including body mass index and neck circumference were measured.* Results*. Significant differences were demonstrated for 12 out of the 19 parameters considered between genders. Uvula and tongue dimensions and the distances between epiglottis-posterior pharyngeal wall and epiglottis-posterior nasal spine were significantly larger in males. The anteroposterior inclination of the uvula and the distances between the uvula and posterior pharyngeal wall were significantly greater in females. No significant differences were found between skeletal classes relative to most of the variables. Body mass index and neck circumference were positively correlated with the dimensions of tongue and uvula.* Conclusions*. Sexual dimorphism relative to some cephalometric variables and anthropometric parameters may account partly for larger oronasopharyngeal spaces in females. Anthropometric data need to be accounted for in population-related comparisons.

## 1. Introduction

Upper airway space (UAS) structures play a significant role in the development of the craniofacial complex and are key parameters in orthodontic diagnosis and treatment planning. In addition, breathing disorders such as obstructive sleep apnea (OSA) are, at least partly, influenced by the craniofacial morphologic features and more specifically the anatomic characteristics of the UAS [1, 2] Currently available evidence suggests that OSA is associated with reduced UAS dimensions [3]. In a cone-beam CT study, Enciso et al. reported that the minimum cross-sectional area of the UAS and its lateral dimension were significantly smaller in OSA patients when compared to snorers [4]. Guilleminault et al. concluded that recurrence of OSA in adolescents considered cured of OSA was associated with a significant reduction of the posterior airway space [5]. Other soft tissue elements of the UAS have also been suggested as significant morphological parameters in OSA such as adenoids, soft palate length, and tongue dimensions [6, 7].

Several studies have investigated whether UAS size is associated with specific craniofacial characteristics using lateral cephalograms [8–11], computed tomography [12], or cone-beam computed tomography (CBCT) [13, 14] in healthy subjects with no pharyngeal or respiratory disturbances. Conflicting results have been reported relative to the correlation between UAS dimensions and sagittal skeletal pattern. While some studies have shown that sagittal skeletal malocclusion affects the UAS size [9, 15], others have failed to demonstrate such association [8, 11, 14]. The discrepancies observed in the abovementioned studies could be attributed to various factors such as sample age [16], gender [17, 18], ethnicity [19], area of measurement (nasopharynx, oropharynx, or hypopharynx), interference related to interrelationship between variables such as vertical and horizontal growth patterns [20], skeletal versus dental classification, inclusion criteria (obesity and smoking) [21], clinical skills and reproducibility in identifying cephalometric landmarks [22], type of measurements (linear, angular, ratio, area, and volume), the use of CBCT versus standard lateral cephalograms [23], head position during imaging [17, 24, 25], and manual versus computer digital tracing [23].

The recognition of ethnic and age- and gender-related differences in the dimensions of the UAS [26, 27] made it necessary to identify reference norms for different populations separately for male and female children and adults [28]. Currently, there is moderate documentation of representative cephalometric characteristics of the UAS in various populations and ethnic groups, which is practically lacking in Middle Eastern populations.

The aims of the present cross-sectional study are to cephalometrically evaluate, in healthy young adult Lebanese subjects, the dimensions of the soft tissue elements of the UAS. A secondary objective is to assess the impact of modifying variables such as gender, skeletal class, and anthropometric parameters on UAS dimensions.

## 2. Materials and Methods

### 2.1. Patient Selection

120 healthy young adults were recruited from the graduate students at the Lebanese University, School of Dentistry, according to the following inclusion criteria:

- Age range 21–25 years; this age group is considered to be at low risk for sleep breathing disorders [29].
- Complete dentitions or at least 3 posterior teeth distal to canines.
- Lebanese origin as confirmed by the family tree and lack of interethnic marriages in the preceding 3 generations.

Exclusion criteria include

- previous diagnosis of sleep breathing disorders; the participants were asked to fill out forms to assess their medical and family history and to answer a series of questions derived from sleep breathing disorders-related questionnaires (Berlin, Epworth Sleepiness Scale, and STOP BANG); all individuals with answers that could indicate any potential sleep breathing disorder were excluded;
- pathologies or morphological anomalies affecting the craniofacial or head and neck structures;
- previous craniofacial or head and neck endoscopies or surgeries;
- oral fixed or removable appliances;
- previous orthodontic treatment;
- professions involving blowing;
- abnormal tongue mobility and tongue thrust.

Prior to data collection, all subjects were informed about the procedures involved in the study and their informed consent was obtained. The study was approved by the Ethical Committee of the Lebanese University (number 7/5/2014).

### 2.2. Cephalometric Analysis

Digital lateral cephalograms were performed in a standardized manner at the Department of Radiology, using Kodak 8000/C apparatus (Carestream Dental, Toronto, Canada). All radiographs were taken by one single clinician using an image field of 195 × 263 mm and matrix dimensions of 1840 × 1360 pixels. The magnification was set at 1 : 1.14. The X-ray tube was positioned at 152.4 cm from the target and the distance of the participant's median plane to the film was set at 18 cm.

Procedures applied during X-ray acquisition were standardized to avoid any impact of posture and functional activity (i.e., breathing and swallowing) on the dimensions of the UAS. Prior to cephalogram acquisition, the subjects were trained through a series of 5–8 exercises to reach a relaxed position of the tongue. Each exercise consisted of 5 consecutive steps: (1) swallowing normally with the mouth closed and the teeth in occlusion; (2) taking a deep breath through the nose; (3) placing the tongue in a relaxed position with the dorsum away from the palate and tip touching the incisal papilla; (4) exhaling slowly from the nose while keeping the teeth in maximal intercuspation as described by Siersbæk-Nielsen and Solow [30] and the tongue away from the palate; (5) holding their breath. The participants were asked to keep their lips in a relaxed position throughout steps (2) to (5).

Once the participants felt comfortable reaching the end point of the exercise, they were positioned standing in the cephalometer and asked not to move their heads from the natural head position [31]. The standing position used was the orthoposition from standing to walking described by Molhave [32]. The subjects were asked to position their tongue in the relaxed position applying the abovementioned steps and signal the correct and complete execution of the exercise to the operator for X-ray acquisition.

The digital cephalograms were transferred into a computer and analyzed using the Viewbox Cephalometric tracing software (Viewbox version 4.0.1.6, 2012, dHAL Software, Kifissia, Greece). Cephalometric soft tissue and skeletal landmarks of the UAS were identified and digitized simultaneously by 2 independent experienced orthodontists blinded to the objectives of the study. Three cephalograms had to be excluded due to poor quality or difficulties in clearly identifying the selected landmarks. Sets of lines, angles, and areas identifying the UAS were traced.

**Figure 1:**
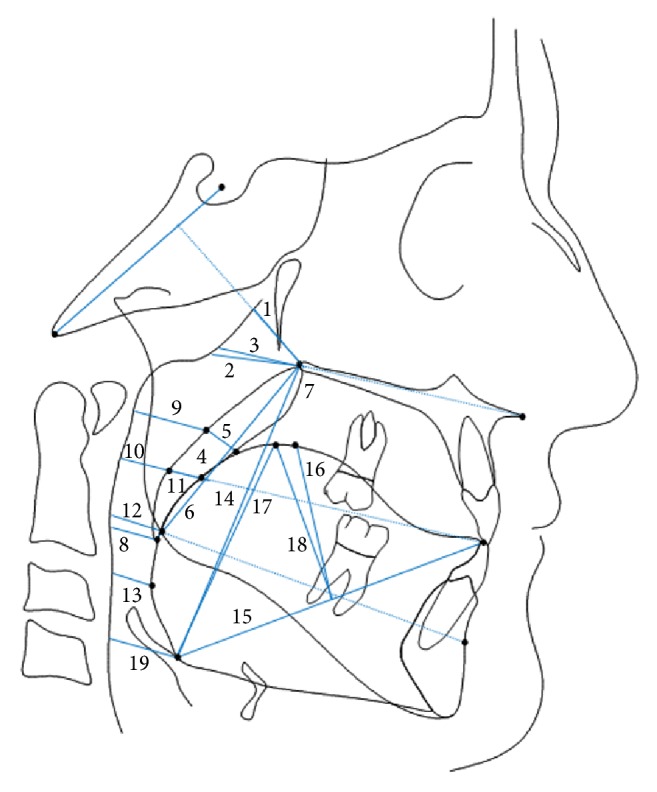
Cephalometric pharyngeal airway space variables divided vertically into (A)* nasopharynx*: (1) PPW Ad2-PNS (distance between posterior nasal spine (PNS) and the posterior pharyngeal wall (PPW) along a line perpendicular to Ba-S), (2) PPW Ad1-PNS (distance between PNS and PPW along the line PNS-Ba), and (3) PPW-PNS (distance between PNS and PPW along the palatal plane); (B)* oropharynx* subdivided into (a)* soft palate and retropalatal areas*: (4) uvula area, (5) uvula thickness (distance between front and back of uvula at its thickest point), (6) PNS-P (soft palate length from PNS to the lower tip of the uvula), (7) ANS-PNS-P angle (formed by the lines ANS-PNS and PNS-P), (8) PPW-P uvula (distance between uvula tip and the PPW along a line parallel to the palatal plane), (9) PPW-uvula dorsum (distance between uvula dorsum (middistance P-PNS) and PPW along a line parallel to the palatal plane), and (10) PPW-UvOp (distance between uvula dorsum and PPW along the occlusal plane); (b)* retroglossal region*: (11) PPW-TgOp (distance between the highest point on tongue dorsum and PPW along the occlusal plane), (12) PPW-GoB (distance between gonion and PPW along the line Go-B), (13) PPW-tongue base (distance between the most posterior point on tongue base and PPW along a line parallel to the palatal plane), and (14) Eb-PNS (vertical airway length); (c)* tongue*: (15) Eb-TT (distance between epiglottis base (Eb) and tongue tip (TT)), (16) TgH (distance between midpoint Eb-TT and the highest point on tongue dorsum), (17) Eb-Tg dorsum (distance between Eb and tongue dorsum), and (18) TgH dorsum (distance between midpoint Eb-TT and tongue dorsum); and (C)* hypopharynx* including (19) PPW-Eb (distance between Eb and PPW along a line parallel to the palatal plane).

Dimensions of the UAS were recorded using 19 variables divided as follows (Figure 1):

- *Nasopharynx* represented by PPW Ad2-PNS, PPW Ad1-PNS, and PPW-PNS.
- *Oropharynx* subdivided in
  - soft palate and retropalatal area represented by uvula area, uvula thickness, PNS-P, ANS-PNS-P angle, PPW-P uvula, PPW-uvula dorsum, and PPW-UvOp;
  - the retroglossal region identified by PPW-TgOp, PPW-GoB, PPW-tongue base, and Eb-PNS;
  - the tongue represented by Eb-TT, TgH, Eb-Tg dorsum, and TgH dorsum.
- *Hypopharynx* defined by PPW-Eb.

For the uvula area measurement, the radiographic borders of the uvula, extending from the posterior nasal spine downward to Point P defined as the tip of the uvula, were identified and the contours marked digitally by the operators using the Viewbox software. The internal area of the uvula was then calculated using the area measurement tool of the same software.

Linear, angular, and surface area measurements were expressed in mm, degrees, and mm^2^, respectively, with the 1 decimal format.

The 2 clinicians had joint calibration exercises for landmark identification and tracing. Subsequently, intra- and interobserver agreement were assessed using one-way intraclass correlation coefficient (ICC) and 95% confidence interval (CI). For this purpose, the 2 clinicians separately performed 3 repeated measurements of all cephalometric variables on 5 cephalograms at 1-week interval. High intraobserver reliability with ICC values of 0.998 and 0.999 was obtained for linear and angular measurements, respectively. ICC values for interobserver reliability were 0.997 and 1.000 with 95% CI (−1.05, 0.50) and (0.17, 1.01) for linear and angular measurements, respectively.

The subjects were classified into 3 skeletal sagittal types according to their ANB angles as class I (1° ≤ ANB ≤ 3°), class II (ANB > 3°), and class III (ANB < 1°).

Anthropometric measurements were performed with the subjects wearing light clothes and without shoes. Height was measured to the nearest 0.01 m using a wall-mounted Seca Bodymeter 208 (Seca®). Body weight was determined to the nearest 0.05 kg with a calibrated digital Tanita® model HD-380 weighting machine (Tanita). Body mass index (BMI) was calculated as weight (kg) divided by the height squared (m^2^). Neck circumference (NC) was measured to the nearest mm with a flexible tape, with the subject in the upright position at the end of gentle expiration, at the level of the cricothyroid membrane (mid thyroid cartilage).

### 2.3. Statistical Analysis

The sample size was set to enable (1) the estimation of any of the cephalometric and anthropometric variables to within a margin of error of at most 0.2 of a standard deviation using 95% confidence intervals and (2) the detection of a moderate to large effect size (0.6 SD and above) difference between any two groups such as males and females. A sample size of 100 participants was calculated accordingly.

Descriptive statistics were performed for all cephalometric and anthropometric variables. After confirmation of the normal sample distribution, paired* t*-test, ANOVA, and Pearson correlation were applied to evaluate the impact of modifying variables (gender, skeletal class, and anthropometric parameters) on UAS characteristics. *p* < 0.05 was set for statistical significance. Statistical analyses were performed using the Statistical Package for the Social Sciences (SPSS for Windows, version 23; SPSS Inc., Chicago, IL, USA).

## 3. Results

Descriptive statistics and comparisons are reported in Tables 1–5. When UAS variables were compared between males and females (Table 1), statistically significant differences were demonstrated for 12 out of the 19 parameters considered. Uvula dimensions (uvula area, uvula thickness, and uvula length PNS-P), tongue length and height (Eb-TT, TgH, TgH dorsum, and Eb-Tg dorsum), and the distances between epiglottis-posterior pharyngeal wall (PPW) and epiglottis-posterior nasal spine were significantly larger in males than in females. The anteroposterior inclination of the uvula represented by the ANS-PNS-P angle and the distances between uvula and PPW (PPW Ad1-PNS and PPW-uvula dorsum) were significantly greater in females.

Comparison between skeletal classes (Table 2) showed no statistically significant differences between classes I and III or between classes I and II relative to any of the 19 variables. The only significant differences were demonstrated between classes II and III relative to the uvula area and uvula thickness with class III individuals showing greater values than class II subjects.

Anthropometric data and gender-related differences are summarized in Table 3. Overall mean BMI was 23.2 ± 3.5 (17.1–32.8). Average figures were 24.6 ± 3.3 (17.6–32.1) and 22.2 ± 3.2 (17.1–32.8) for males and females, respectively. NC ranged from 30.5 to 56.6 cm (38.1 ± 3.6 cm) and from 28.1 to 57.2 cm (31.4 ± 3.4 cm) in males and females, respectively. All anthropometric measurements were significantly greater in males than in females (Table 3).

Gender-related differences in the 12 abovementioned UAS parameters remained significant after adjusting for differences in BMI between males and females.

Skeletal classes with their relative differences in anthropometric data are presented in Table 4.

There were no statistically significant differences in BMI between the three classes while body weight, body height, and NC were significantly greater in class III when compared to class II. Uvula (area, thickness, and length PNS-P) and tongue dimensions (length Eb-TT and height represented by TgH, TgH dorsum, and Eb-Tg dorsum) were positively correlated with BMI and NC (Table 5). A significant positive correlation was also found between the vertical airway length of the pharynx (Eb-PNS) and both BMI and NC. The sagittal width of the UAS at the epiglottis base (PPW-Eb) was positively correlated with NC. Negative correlations were found between parameters defining the sagittal width of the UAS at the levels of posterior nasal spine (PPW-Ad1-PNS), uvula dorsum (PPW-uvula dorsum), and both BMI and NC.

## 4. Discussion

The present study evaluated UAS dimensions in young adult Lebanese subjects. Although it did not include matched samples of other ethnic groups or neighboring populations, indirect comparisons can be tentatively made between the present Lebanese data and the results of previously published cephalometric investigations conducted in young adult healthy individuals and using lateral cephalometric imaging. The shortest distance between the soft palate and the PPW (PPW-P uvula) in the Lebanese sample averaged 10.5 mm which matches previously published figures in White Brazilians [8] and Caucasians [26, 33] where values of 9-10 mm were reported. The smallest anteroposterior width of the UAS located along the plane GoB (PPW-GoB) was 6.9 ± 4.7 mm in the present study. This value does not fall within the range of average measurements (10–12 mm) found in the abovementioned studies and is smaller than the figures reported in other ethnic groups such as Blacks and Hispanics (9.0 ± 3.6 mm and 9.3 ± 4.6 mm, resp.) [26]. When compared with Caucasians, Blacks, and Hispanics [26], the investigated Lebanese population seems to have a more reduced soft palate length (PNS-P) (37.4 ± 4.6 mm versus 44.1 ± 5.6 mm in Caucasians, 46.2 ± 4.7 mm in Blacks, and 42.8 ± 6.6 mm in Hispanics). Conversely, soft palate length and uvula thickness in the present study (37.4 mm and 9.7 mm, resp.) are greater than those reported in Indians (30.9 mm and 7.9 mm, resp.) [34]. While some of the abovementioned discrepancies could be related to sample characteristics (i.e., anthropometric data, smoking, etc.) [21], reproducibility in identifying cephalometric landmarks between different investigators, and imaging technique protocols [17, 23–25], the contrast may possibly implicate population-related differences.

In the present study, 10 anteroposterior measurements from the PPW to the soft and hard tissue structures located anteriorly were evaluated at different levels (PPW Ad2-PNS, PPW Ad1-PNS, PPW-PNS, PPW-P uvula, PPW-uvula dorsum, PPW-UvOp, PPW-TgOp, PPW-GoB, PPW-tongue base, and PPW-Eb) with PPW-GoB showing the smallest mean value when compared to the other 9 measurements. The smallest section of the UAS is of the greatest relevance clinically because the conductance of respiratory gases is dictated by its narrowest part. In the published literature, different levels and reference points were used to define the smallest width of the airway space behind the tongue. Martin et al. [15] used McNamara's lower pharynx dimension defined as the minimum distance between the point where the posterior tongue contour crosses the mandible and the nearest point on the PPW while other authors [35] used the true minimal airway space dimension. PPW-GoB referred to as PAS (posterior airway space) or IAS (inferior airway space) was also applied to define the shortest distance of the airway along a line through Go to B-point [36]. Although PPW-GoB may not correspond to the true smallest width of the airway space depending on the existing anatomy, it has been significantly correlated with the smallest volume measured behind the base of the tongue [37]. In conclusion, discrepancies in defining the shortest dimension of the pharyngeal space in the retroglossal area render any interpretation and comparison of previously published results difficult.

This study demonstrated significant differences between genders related to most UAS-related parameters. Sexual dimorphism is a usual finding in investigations evaluating pharyngeal morphometry in healthy young adults [15, 27, 34, 35]. The variables related to the nasopharynx showed a trend towards larger values in females although statistical significance was reached only for PPW Ad1-PNS. Lebanese males tend to have larger soft tissues in the oropharynx area (soft palate and tongue) than females, in agreement with findings in other populations [27, 34, 35]. However, this was not coupled with significant differences in the sagittal linear measurements of the UAS (PPW-P uvula, PPW-UvOp, PPW-TgOp, PPW-GoB, and PPW-tongue base). This conclusion is consistent with the findings in young adult European Spanish [15] and Chinese [35] populations, where sexual dimorphism in the nasopharynx was not associated with gender-related differences in the minimum depth of the airway (corresponding to the measurement PPW-GoB of the present study). It should be noted that the statistically significant differences for 12 out of the 19 cephalometric UAS variables between genders could be partly attributed to anthropometric differences since BMI was significantly greater in males when compared to females. Whether gender-related differences in the dimensions of the naso- and oropharyngeal spaces are inherent to different anatomy or to anthropometric data needs to be further investigated.

Although the angle formed by the soft palate and the palatal plane showed greater values in Lebanese females than in males, this did not result in a more reduced pharyngeal space posteriorly to the uvula. In fact, the linear dimension PPW-uvula dorsum was greater in females and the other 2 uvula-related airway dimensions located more inferiorly (PPW-UvOp and PPW-TgOp) were not different between genders. This finding emphasizes the importance of uvula thickness as a key variable in dictating the sagittal dimensions of the UAS.

Several cephalometric and CBCT studies [8, 11, 14, 38] reported that sagittal class does not appear to influence UAS dimensions both in children [8, 11, 38] and in adults [14]. Conversely, other investigations highlighted the variability of UAS dimensions in different sagittal skeletal relationships in children [13, 38–42] and adults [9, 12, 15, 18, 35, 43]. In the present study, significant differences were only demonstrated between class II and class III subjects relative to uvula area and thickness with class III patients presenting greater uvula dimensions. The abovementioned discrepancies are likely to be associated with differences in sample anthropometric characteristics, technical differences, and population-related variability. The increased uvula dimensions in the present investigation might be associated with the larger relative number of males in the class III group (15 males versus 5 females) and their lower relative number in the class II group (20 males versus 47 females). In addition, the larger NC in the class III subjects might account partly for the greater uvula dimensions in this group.

Within the last decade, 3D imaging such as cone-beam computed tomography (CBCT) became more frequently incorporated in orthodontic diagnosis and treatment. Despite the significant advantages of this technology related to the possibility of obtaining volumetric assessments and cross-sectional evaluations at multiple levels, its wide application in large-scale studies is still hindered by factors such as cost and availability. CBCT exposes patients to greater radiation doses than conventional lateral cephalograms [44–47] and therefore its application in the context of cross-sectional studies is not ethically justifiable. Furthermore, conventional lateral cephalograms and those derived from 3D CBCTs demonstrated no significant differences in most linear and angular cephalometric measurements [48–50] and more specifically in the UAS area [51]. Linear 2D cephalometric measurements relate well to three-dimensional magnetic resonance imaging measurements [52]. Riley and Powell [53] reported a high correlation (*r* = 0.92) between posterior airway space on cephalometric radiographs and the volume of the pharyngeal airway on CT scans. However, it should be highlighted that 2D conventional lateral cephalograms have limitations in representing 3D structures due to distortion, differences in magnifications, and superimposition of bilateral structures [54]. Airway parameters derived from conventional lateral cephalograms have not been demonstrated to consistently predict the 3D volumes of UAS and related soft tissue structures [55, 56] while CBCT is effective and reliable in assessing such variables [54, 57–60]. Further studies are needed to validate the findings of the present study using CBCT-generated lateral cephalograms including additional airway and soft tissue volumetric parameters.

Several investigators have highlighted airway soft tissue changes that occur with postural change from upright to supine positions [61] and reduction in the posterior airway space behind the soft palate [62]. Therefore, it seems that supine cephalometry might be more relevant clinically to assess the upper airways and related structures in sleep breathing disorders. In the present investigation, the impact of modifying variables on airway space parameters was evaluated in healthy individuals under standardized postural and dynamic conditions with the head stabilized in a cephalostat to eliminate position-related variations. In addition, the figures obtained in the present study can be compared, though indirectly, to results from most of the currently available published norms of various ethnic groups/populations which are still largely based on readings in the upright position. It remains however difficult to extend gender- and BMI-dependent variations of the UAS dimensions to participants in the supine posture. More well-designed trials are needed to validate such hypothesis.

In the present study, BMI had a significant impact on some UAS characteristics such as adenoid-related measurements (PPW Ad2-PNS, PPW Ad1-PNS), uvula (uvula length PNS-P, thickness, and area) and tongue dimensions (length Eb-TT, height TgH, TgH Dorsum, and Eb-Tg Dorsum), posterior airway space dimensions at the levels of the uvula (PPW-uvula dorsum, PPW-UvOp), and vertical airway length (Eb-PNS). Larger tongues and uvula were present in subjects with greater BMI and were associated with a reduction of the retropalatal area. While no direct measurement of the adenoid mass has been performed, the linear distances from the posterior nasal spine to the adenoid in 2 different planes (PPW Ad2-PNS, PPW Ad1-PNS) were reduced, indicating potential changes in the adenoid size with increasing BMI. This finding has not been previously documented in healthy subjects and warrants further investigations to better understand the mechanisms by which BMI affects the lymphoid tissue dimensions in health.

A significant association was also observed when NC was correlated with UAS variables. These findings are in line with the conclusions of several investigations correlating UAS dimensions with BMI and NC in healthy adults without UAS pathologies [63]. Such correlations are extremely important to account for in ethnic- and population-related comparisons of UAS dimensions as they can be incriminated in the differences demonstrated between ethnic groups. Studies specifically designed to detect population-related differences should apply matched anthropometric data to comparative groups in order to identify true differences.

It is interesting to note that the retroglossal dimensions of the UAS were not affected by larger tongues in the presence of higher BMI values. This can be attributed to the fact that fat deposition increases tongue dimensions (length Eb-TT, height TgH, TgH Dorsum, and Eb-Tg Dorsum) but not necessarily its posterior projection into the retroglossal area. While the impact of larger tongues on the restriction of the retroglossal space is not evident in the upright position, it is likely to be more significant in the supine posture due to the sagging of the base of the tongue against the PPW, gravitational pull [64, 65], and the hypotonicity of the genioglossal muscle during sleep [66].

## 5. Conclusions

Within the limitations of this study, gender and anthropometric characteristics seem to have a significant influence on most UAS dimensions in healthy young adult Lebanese individuals while sagittal skeletal class had no such impact.

Sexual dimorphism may, at least partly, account for larger naso- and oropharyngeal spaces in females. It is difficult to attribute this finding to differences in the anatomical structures of the UAS or to anthropometric variations between genders.

Further larger scale comparative studies including matched modifying variables such as gender, skeletal class, BMI, and other anthropometric data are needed to assess similarities and differences between Lebanese and other population groups.

## Figures and Tables

**Table 1:** Descriptive analysis of the selected cephalometric pharyngeal airway space variables in the sample population and comparison between genders. *∗* refers to statistically significant differences between genders.

Variables	Males (*n* = 48)	Females (*n* = 69)	*p *value	Overall (*n* = 117)
	Mean ± SD	Mean ± SD		Mean ± SD
Nasopharynx				
Adenoid				
PPW Ad2-PNS (mm)	23.6 ± 3.7	24.9 ± 3.4	0.070	24.4 ± 3.6
PPW Ad1-PNS (mm)	27.3 ± 3.1	28.8 ± 3.4	0.011^*∗*^	28.2 ± 3.3
PPW-PNS (mm)	28.8 ± 2.9	29.6 ± 3.3	0.178	29.2 ± 3.2
Oropharynx				
Soft palate and retropalatal area				
Uvula area (mm^2^)	315.4 ± 68.5	243.4 ± 45.1	<0.001^*∗*^	272.3 ± 65.9
Uvula thickness (mm)	10.6 ± 2.4	9.0 ± 1.3	<0.001^*∗*^	9.7 ± 2.0
PNS-P (mm)	39.4 ± 4.4	36.0 ± 4.2	<0.001^*∗*^	37.4 ± 4.6
ANS-PNS-P angle (degree)	130.2 ± 6.4	132.8 ± 5.8	0.029^*∗*^	131.7 ± 6.1
PPW-P uvula (mm)	10.2 ± 2.7	10.7 ± 2.9	0.297	10.5 ± 2.8
PPW-uvula dorsum (mm)	11.00 ± 4.0	13.5 ± 3.8	0.001^*∗*^	12.5 ± 4.0
PPW-UvOp (mm)	8.8 ± 3.1	9.8 ± 3.0	0.095	9.4 ± 3.1
Retroglossal				
PPW-TgOp (mm)	22.5 ± 16.3	19.4 ± 9.0	0.233	20.7 ± 12.5
PPW-GoB (mm)	6.4 ± 4.8	7.4 ± 4.6	0.300	7.0 ± 4.6
PPW-tongue base (mm)	11.9 ± 4.6	11.8 ± 4.5	0.940	11.8 ± 4.5
Eb-PNS (mm)	79.3 ± 6.5	67.0 ± 6.4	<0.001^*∗*^	72.0 ± 8.8
Tongue				
Eb-TT (mm)	88.0 ± 6.3	80.9 ± 5.5	<0.001^*∗*^	83.8 ± 6.8
TgH (mm)	42.6 ± 5.2	37.0 ± 4.4	<0.001^*∗*^	39.2 ± 5.4
Eb-Tg dorsum (mm)	73.7 ± 9.3	64.7 ± 6.5	<0.001^*∗*^	68.3 ± 8.9
TgH dorsum (mm)	44.5 ± 5.4	37.9 ± 4.9	<0.001^*∗*^	40.6 ± 6.0
Hypopharynx				
Epiglottis				
PPW-Eb (mm)	22.6 ± 9.4	18.5 ± 4.2	0.002^*∗*^	20.2 ± 7.1

**Table 2:** Descriptive analysis of the selected cephalometric pharyngeal airway space variables in each of the 3 skeletal groups without gender differences. M: males; F: females; NS: nonsignificant differences. *∗* refers to statistically significant differences between classes.

Variables	Class I (M = 13; F = 17)	Class II (M = 20; F = 47)	*p *value	Class II (M = 20; F = 47)	Class III (M = 15; F = 5)	*p *value	Class I (M = 13; F = 17)	Class III (M = 15; F = 5)	*p *value
	Mean ± SD	Mean ± SD		Mean ± SD	Mean ± SD		Mean ± SD	Mean ± SD	
Nasopharynx									
Adenoid									
PPW Ad2-PNS (mm)	25.0 ± 3.9	24.1 ± 3.3	NS	24.1 ± 3.3	24.1 ± 4.2	NS	25.01 ± 3.85	24.1 ± 4.2	NS
PPW Ad1-PNS (mm)	28.6 ± 3.4	28.1 ± 3.4	NS	28.1 ± 3.4	28.1 ± 2.9	NS	28.57 ± 3.43	28.1 ± 3.0	NS
PPW-PNS (mm)	29.7 ± 3.0	29.1 ± 3.2	NS	29.1 ± 3.2	29.2 ± 3.1	NS	29.69 ± 3.00	29.2 ± 3.1	NS
Oropharynx									
Soft palate and retropalatal area									
Uvula area (mm^2^)	275.9 ± 73.6	260.5 ± 58.4	NS	260.5 ± 58.4	308.6 ± 68.8	0.017^*∗*^	275.9 ± 73.6	308.6 ± 68.8	NS
Uvula thickness (mm)	10.6 ± 2.4	9.2 ± 1.6	NS	9.2 ± 1.6	10.5 ± 2.2	0.013^*∗*^	10.2 ± 2.4	10.5 ± 2.3	NS
PNS-P (mm)	36.4 ± 4.5	37.4 ± 4.5	NS	37.4 ± 4.5	38.8 ± 4.8	NS	36.4 ± 4.5	38.8 ± 4.8	NS
ANS-PNS-P angle (degree)	132.2 ± 6.0	131.6 ± 5.9	NS	131.6 ± 5.9	131.3 ± 7.3	NS	132.1 ± 6.0	131.3 ± 7.3	NS
PPW-P uvula (mm)	10.9 ± 2.7	10.4 ± 3.0	NS	10.4 ± 3.0	10.6 ± 2.5	NS	10.9 ± 2.8	10.6 ± 2.5	NS
PPW-uvula dorsum (mm)	12.8 ± 4.2	12.7 ± 3.9	NS	12.7 ± 3.9	11.5 ± 4.5	NS	12.8 ± 4.2	11.5 ± 4.5	NS
PPW-UvOp (mm)	9.8 ± 3.0	9.4 ± 3.1	NS	9.4 ± 3.1	8.7 ± 3.1	NS	9.8 ± 3.0	8.7 ± 3.1	NS
Retroglossal									
PPW-TgOp (mm)	24.2 ± 18.3	19.5 ± 10.3	NS	19.5 ± 10.3	20.3 ± 7.5	NS	24.2 ± 18.3	20.3 ± 7.5	NS
PPW-GoB (mm)	7.6 ± 4.8	6.6 ± 4.7	NS	6.6 ± 4.7	7.5 ± 4.2	NS	7.6 ± 4.8	7.5 ± 4.2	NS
PPW-tongue base (mm)	11.0 ± 4.2	11.6 ± 4.0	NS	11.6 ± 4.1	14.0 ± 5.9	NS	11.0 ± 4.2	14.0 ± 5.9	NS
Eb-PNS (mm)	71.6 ± 9.6	71.3 ± 8.2	NS	71.3 ± 8.2	75.2 ± 9.5	NS	71.6 ± 9.6	75.2 ± 9.5	NS
Tongue									
Eb-TT (mm)	83.9 ± 6.0	83.7 ± 6.5	NS	83.7 ± 6.4	85.1 ± 8.0	NS	83.9 ± 6.0	85.1 ± 8.0	NS
TgH (mm)	39.2 ± 4.7	38.9 ± 5.9	NS	38.9 ± 5.9	40.6 ± 4.7	NS	39.2 ± 4.7	40.6 ± 4.7	NS
Eb-Tg dorsum (mm)	66.8 ± 8.6	68.5 ± 8.7	NS	68.5 ± 8.7	70.0 ± 10.1	NS	66.8 ± 8.6	70.0 ± 10.1	NS
TgH dorsum (mm)	40.1 ± 5.7	40.5 ± 6.3	NS	40.5 ± 6.3	41.8 ± 5.6	NS	40.1 ± 5.6	41.8 ± 5.6	NS
Hypopharynx									
Epiglottis									
PPW-Eb (mm)	21.9 ± 12.0	19.2 ± 4.2	NS	19.2 ± 4.2	21.2 ± 5.1	NS	21.9 ± 12.0	21.2 ± 5.1	NS

**Table 3:** Anthropometric data in 117 young adult healthy Lebanese subjects. *∗* refers to significant differences between males and females.

Anthropometric measurements	Males (*n* = 48)	Females (*n* = 69)	*p* value	Overall (*n* = 117)
	Mean ± SD	Mean ± SD		Mean ± SD
Body weight (kg)	76.1 ± 12.4	57.7 ± 8.1	<0.001^*∗*^	65.2 ± 13.5
Body height (cm)	175.7 ± 7.6	161.5 ± 6.0	<0.001^*∗*^	167.3 ± 9.7
Body mass index (kg/m^2^)	24.6 ± 3.3	22.2 ± 3.2	<0.001^*∗*^	23.2 ± 3.5
Neck circumference (cm)	38.1 ± 3.6	31.4 ± 3.4	<0.001^*∗*^	34.2 ± 4.8

**Table 4:** Anthropometric data in the different skeletal classes. *∗* refers to significant differences between class II and class III.

Anthropometric measurements	Class I	Class II	Class III	*p* value
	Mean ± SD	Mean ± SD	Mean ± SD	
Body weight (kg)	65.95 ± 14.17	62.91 ± 12.12	72.38 ± 15.05	0.021^*∗*^
Body height (cm)	168.02 ± 9.47	165.48 ± 8.97	172.53 ± 10.86	0.014^*∗*^
Body mass index (kg/m^2^)	23.22 ± 3.78	22.88 ± 3.27	24.10 ± 3.45	0.383
Neck circumference (cm)	33.82 ± 3.39	33.48 ± 4.66	36.90 ± 6.07	0.016^*∗*^

**Table 5:** Correlation between anthropometric and airway variables. ^*∗*^Correlation is significant at the 0.05 level (2-tailed). ^*∗∗*^Correlation is significant at the 0.01 level (2-tailed).

Variables	Pearson correlation	Neck circumference	Body mass index
Nasopharynx			
Adenoid			
PPW Ad2-PNS (mm)	Pearson correlation	−0.156	−.234^*∗*^
	Sig. (2-tailed)	0.095	0.011
PPW Ad1-PNS (mm)	Pearson correlation	−.185^*∗*^	−.240^*∗∗*^
	Sig. (2-tailed)	0.046	0.009
PPW-PNS (mm)	Pearson correlation	−0.048	−0.110
	Sig. (2-tailed)	0.605	0.237
Oropharynx			
Soft palate and retropalatal area			
Uvula area (mm^2^)	Pearson correlation	.450^*∗∗*^	.437^*∗∗*^
	Sig. (2-tailed)	0.000	0.000
Uvula thickness (mm)	Pearson correlation	.320^*∗∗*^	.284^*∗∗*^
	Sig. (2-tailed)	0.001	0.002
PNS-P (mm)	Pearson correlation	.329^*∗∗*^	.313^*∗∗*^
	Sig. (2-tailed)	0.000	0.001
ANS-PNS-P angle (degree)	Pearson correlation	−0.095	0.020
	Sig. (2-tailed)	0.315	0.831
PPW-P uvula (mm)	Pearson correlation	−0.049	−.199^*∗*^
	Sig. (2-tailed)	0.602	0.033
PPW-uvula dorsum (mm)	Pearson correlation	−.189^*∗*^	−.379^*∗∗*^
	Sig. (2-tailed)	0.044	0.000
PPW-UvOp (mm)	Pearson correlation	−0.101	−.292^*∗∗*^
	Sig. (2-tailed)	0.308	0.003
Retroglossal			
PPW-TgOp (mm)	Pearson correlation	0.107	−0.074
	Sig. (2-tailed)	0.252	0.429
PPW-GoB (mm)	Pearson correlation	−0.053	0.158
	Sig. (2-tailed)	0.571	0.088
PPW-tongue base (mm)	Pearson correlation	0.011	0.057
	Sig. (2-tailed)	0.905	0.545
Eb-PNS (mm)	Pearson correlation	.519^*∗∗*^	.245^*∗∗*^
	Sig. (2-tailed)	0.000	0.007
Tongue			
Eb-TT (mm)	Pearson correlation	.423^*∗∗*^	.321^*∗∗*^
	Sig. (2-tailed)	0.000	0.000
TgH (mm)	Pearson correlation	.430^*∗∗*^	.279^*∗∗*^
	Sig. (2-tailed)	0.000	0.003
Eb-Tg dorsum (mm)	Pearson correlation	.436^*∗∗*^	.270^*∗∗*^
	Sig. (2-tailed)	0.000	0.004
TgH dorsum (mm)	Pearson correlation	.467^*∗∗*^	.316^*∗∗*^
	Sig. (2-tailed)	0.000	0.001
Hypopharynx			
Epiglottis			
PPW-Eb (mm)	Pearson correlation	.203^*∗*^	0.001
	Sig. (2-tailed)	0.028	0.988
